# The prognostic importance of cystatin C in severe systolic dysfunction without chronic kidney disease

**DOI:** 10.4103/0256-4947.72280

**Published:** 2010

**Authors:** Serkan Ordu

**Affiliations:** Duzce University, Duzce, Turkey orduserkan@yahoo.com

**To the Editor**: Cystatin C has been identified as a novel biomarker that is more sensitive in detecting early kidney dysfunction. We aimed to investigate the prognostic importance of cystatin C in stable heart failure patients who had an ejection fraction (EF) of <35% and a glomerular filtration rate (GFR) of >60 mL/min/ 1.73 m^2^. Seventy-five stable heart failure patients (50 males and 25 females with a mean age (SD) of 67.6 (10.6) were included in this study. All patients were evaluated using Doppler echocardiography, and biochemical variables including measurement of cystatin C were measured at baseline. Patients were prospectively followed-up for 13(1) months. The endpoints were all-cause mortality and major cardiac adverse events (MACE), which were mortality and rehospitalization. Cystatin C serum levels were measured using a sandwich enzyme immunoassay (ELISA) kit (Biovendor Research and Diagnostic Products, www.biovendor.com).

Serum levels of cystatin C ranged from 0.47 to 2.02 ng/mL (median 1.34 [0.42] ng/mL). Eleven patients died and 34 MACE occurred during follow-up. Mean cystatin C level was significantly higher in the participants who died or had MACE. Cystatin C was significantly correlated with serum creatinine level (*P*=.001, r=0.368) and NYHA class (*P*=.02, r=0.25). Mean high-sensitivity C reactive protein (hsCRP, NYHA class, uric acid and troponin I were significantly higher and hemoglobin level was lower in the mortality group (**Table 1**). Creatinine and GFR were similar between the patients who survived or had MACE. Echocardiographic parameters and biochemical variables (except total cholesterol) were not statistically different. Logistic regression analysis revealed that only cystatin C level was an independent risk factor for MACE rate (hazard ratio=32.56, 95% confidence interval, 2.26-468.62, *P*=.01). For mortality rate, the same analyses revealed an independent risk with cystatin C, which did not reach significance (OR: 37.1, 95% CI, 0.94-1464, *P*=.054). According to ROC analysis, cystatin C levels >1.55 ng/mL could predict mortality with 82% sensitivity and 73% specificity. Furthermore, cystatin C levels >1.45 ng/mL could predict MACE rate with 76% sensitivity and 83% specificity. Kaplan-Meier analysis showed that patients who had serum cystatin C levels of >1.5 ng/mL had a significantly higher total death rate compared with lower levels (*P*<.001).

**Table 1:** Clinical characteristics of survivors and patients who were free of major cardiac adverse events (MACE) and characteristics of patients who died or had MACE during follow up.

	Survivors (n=64)	Mortality group(n=11)	*P*
Age (year)	67.2 (10.8)	70.0 (9.4)	.79
NYHA class III	16	6	.047
Ejection fraction	29.0 (5.6)	30.5 (6.4)	.31
hsCRP (mg/dl)	1.09 (1.11)	3.89 (3.18)	.03
Hemoglobin(g/dl)	13.0 (1.6)	11.6 (1.8)	.001
GFR (mL/min per 1.73 m^2^)	-	-	
Creatinine (mg/dl)	0.91 (0.22)	1.04 (0.17)	.075
Cystatin C (ng/mL)	1.27 (0.41)	1.71 (0.21)	.016
Uric acid (mg/dl)	6.0 (1.9)	7.8 (2.2)	.009
sTroponin levels (μg/dl)	0.08 (0.27)	0.44 (0.88)	.02

	**MACE free (n=41)**	**MACE (n=34)**	***P***

Hemoglobin (g/dl)	13.3 (1.6)	12.3 (1.7)	.01
GFR (mL/min per 1.73 m^2^)	79.4 (17.0)	81.7 (22.2)	.63
CystatinC (ng/mL)	1.15 (0.37)	1.55 (0.35)	<.001
High sensitive CRP (mg/dl)	0.98 (0.87)	2.1 (2.45)	.023
Troponin I (μg/dl)	0.031 (0.17)	0.29 (0.62)	.02

Values are mean (standard deviation).

Cystatin C is a small serine protease inhibitor that is secreted from almost all active cells in the body. It has been identified as a novel biomarker that is more sensitive in detecting early kidney dysfunction compared with creatinine and creatinine-based estimated GFR.^1^ Previous studies dealing with the prognostic value of cystain C in heart failure were conducted on patients with EF>40%.^1^,^2^ Our results showed further insight about the prognostic value of cystatin C in stable heart failure patients who had lower ejection fraction (<35%). The results of the Cardiovascular Health Study showed that elevated cystatin C concentrations were associated with incrementally increasing mortality risk, particularly over 1.0 ng/mL.^3^ The results of the study confirm the same result since none of the patients in our study group having a baseline cystatin C level of < 1 ng/mL died during follow-up.
